# The Nevus Lipomatosus Superficialis of Face: A Case Report and Literature Review

**DOI:** 10.1055/a-2222-1226

**Published:** 2024-01-24

**Authors:** Jae-Won Yang, Mi-Ok Park

**Affiliations:** 1 Gangnam Jaejun Plastic Clinic, Pyeongtaek-si, Gyeongi-do, Korea; 2 MioGreensumer Pathology Clinic, Incheon City, Korea

**Keywords:** nevus lipomatosus superficialis, sebaceous trichofolliculoma, *Demodex*, hamartoma

## Abstract

Nevus lipomatosus superficialis (NLS) is a hamartoma of adipose tissue, rarely reported in the past 100 years. We treated one case, and we conducted a systematic review of the literature. A 41-year-old man presented with a cutaneous multinodular lesion in the posterior region near the right auricle. The lesion was excised and examined histopathologically. To review the literature, we searched PubMed with the keyword “NLS.” The search was limited to articles written in English and whose full text was available. We analyzed the following data: year of report, nation of corresponding author, sex of patient, age at onset, duration of disease, location of lesion, type of lesion, associated symptoms, pathological findings, and treatment. Of 158 relevant articles in PubMed, 112 fulfilled our inclusion criteria; these referred to a total of 149 cases (cases with insufficient clinical information were excluded). In rare cases, the diagnosis of NLS was confirmed when the lesion coexisted with sebaceous trichofolliculoma and *Demodex* infestation. Clinical awareness for NLS has increased recently. NLS is an indolent and asymptomatic benign neoplasm that may exhibit malignant behavior in terms of huge lesion size and specific anatomical location. Early detection and curative treatment should be promoted.

## Introduction

Nevus lipomatosus superficialis (NLS) is a benign cutaneous hamartoma in which mature adipose tissue is deposited ectopically between collagen bundles in the dermal layer, as observed on histopathological examination. It was first reported by Hoffmann and Zurhelle in 1921. 1 The lesions have two clinical manifestations: (1) The classical type is a zosteriform pattern consisting of nontender, soft, skin-colored or yellow papules, nodules, or plaques that are present at birth or appear during the first three decades of life, and (2) the solitary type is a single sessile, dome-shaped papule or nodule that appears in the third to sixth decades of life. 2 Malignant transformation has not been reported. Surgical excision is curative, and no recurrence has been reported.

We describe a case of NLS that manifested with an unusual combination of histopathological findings of sebaceous trichofolliculoma and *Demodex* infestation. We then discuss the findings of a literature review and summarize the clinical manifestations.

## Case

**Fig. 1:**
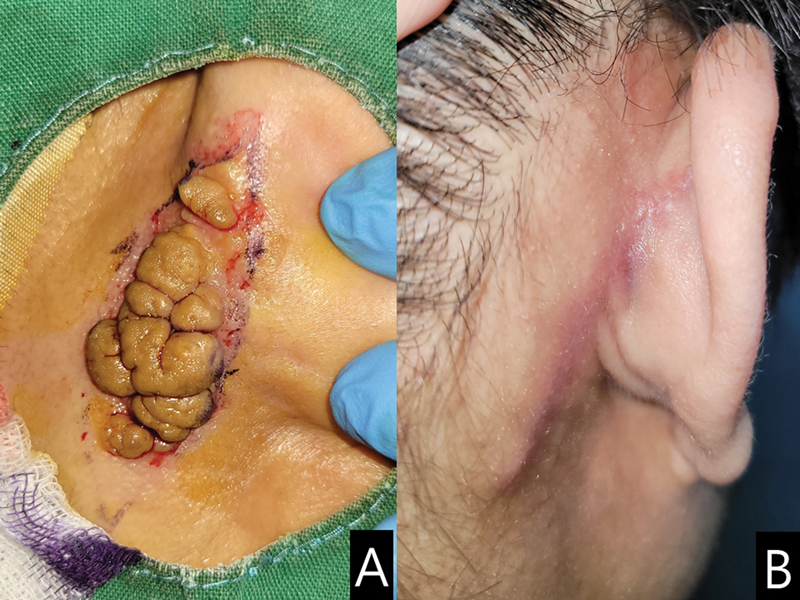
Preoperative appearance. The lesion (3 × 1.5 cm) was noted in the posterior region near the right auricle over the auriculotemporal sulcus. **(A)** An elliptical excision line was designed. **(B)** After surgery, the skin margins of the excised wound were approximated and well healed by postoperative day 15.

The patient, a 41-year-old man, presented with a 35 × 20 mm, cerebriform nodular skin lesion in the posterior region near the right auricle, and satellite papules on the posterior surface of the auricle; these asymptomatic lesions had been present since the age of 14 years. The lesions became prominent after the age of 16 years and gradually enlarged after the age of 29 years. The patient sought treatment for cosmetic reasons alone. Only the main lesion, including the subcutaneous tissue, was excised elliptically. To achieve approximation of both skin margins, the skin flaps were advanced after the undermining procedure. Preoperative and postoperative findings are illustrated in Fig. 1 . By 3 months after surgery, the lesion had not recurred.

### Gross and Histopathological Findings

**Fig. 2:**
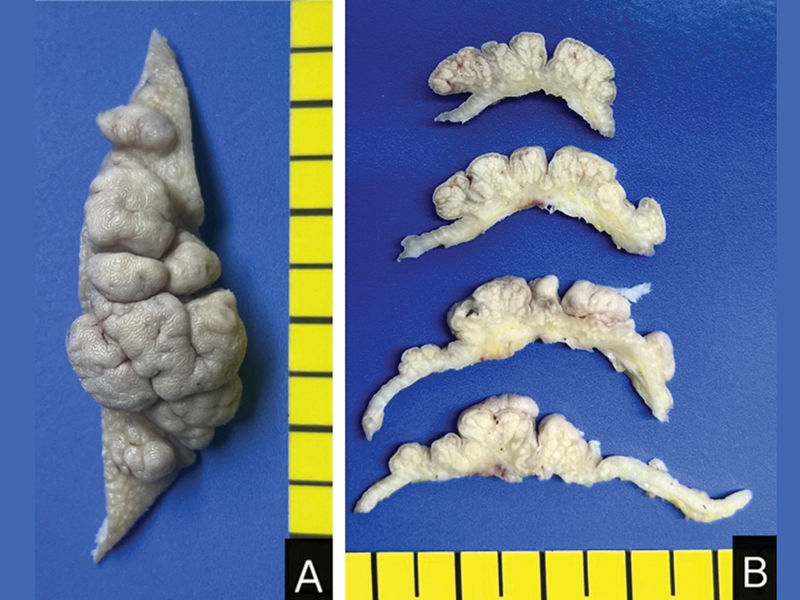
Gross photographs of serial sections, showing skin-colored pedunculated multiple nodules with cerebriform surfaces **(A)** and yellowish-white lobulated nodules and underlying adipose tissue in the dermis **(B)** .

**Fig. 3:**
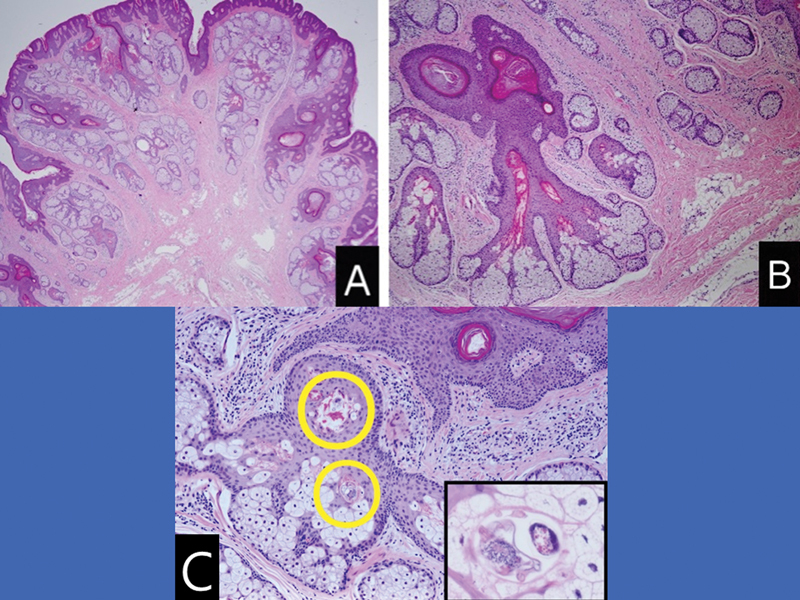
The nodule was composed of many sebaceous lobules with hair follicles **(A)** and underlying groups of mature fat cells among the dermal collagen **(B)** . Hematoxylin and eosin ([H&E]; magnifications, 12.5 **[A]** and 40 **[B]** ). **(C)** *Demodex* mites were found in follicles with perifollicular inflammation and in sebaceous glands (inset) (H&E; magnifications, 100 **[C]** and 400 [inset]).

The excised skin specimen (52 × 23 mm) contained a 25 × 25 skin-colored nodule with a cerebriform surface. Serial sectioning revealed yellowish-white contents, lobulation, and deposition of adipose tissue in the dermis ( Fig. 2 ). Histopathological examination revealed that the nodule was composed of folliculosebaceous material and that groups of mature adipose tissue occupied the whole dermis ( Fig. 3A, B ). *Demodex* mites were also present in follicles and sebaceous glands with perifollicular inflammation ( Fig. 3C ). The pathological diagnosis was NLS with sebaceous trichofolliculoma and *Demodex* infestation with inflammation.

### Literature Review

**Fig. 4:**
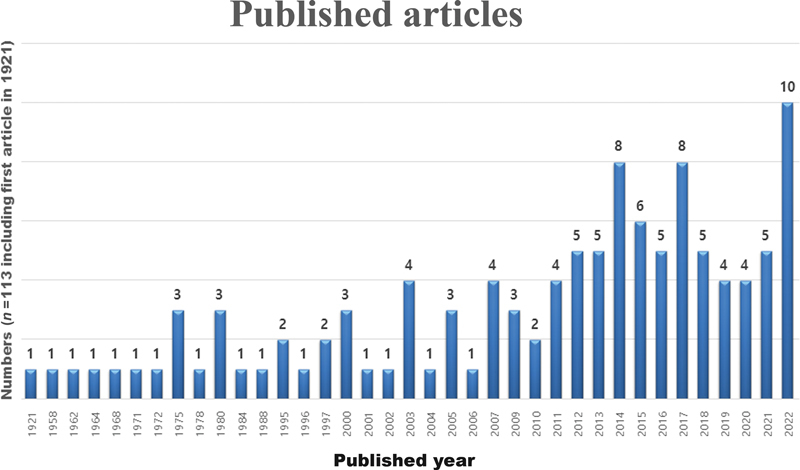
A case of nevus lipomatosus superficialis (NLS) was first reported in 1921. We analyzed 112 articles identified in the PubMed search. The number of reports of NLS has increased gradually since 2003.

**Fig. 5:**
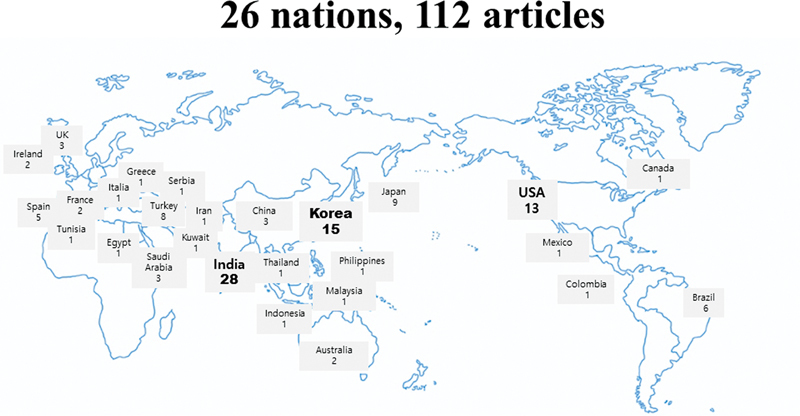
The 112 selected articles concerned cases reported from all continents and 26 nations. We found no geographic preponderance.

**Fig. 6:**
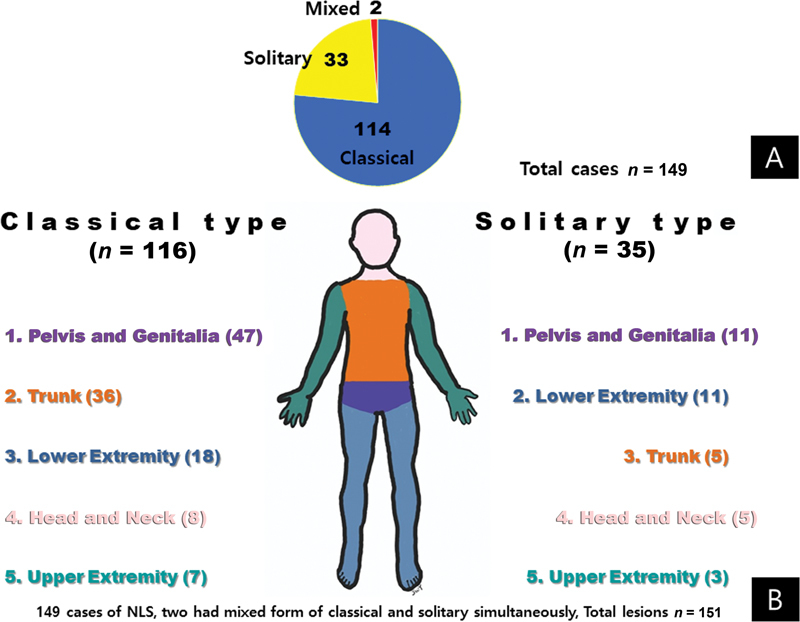
Nevus ipomatosus superficialis (NLS) has two manifestations: classical and solitary. **(A)** Among the selected articles, the classical type was reported more commonly than the solitary type. Two patients had both classical and solitary lesions. **(B)** The lesions were located in five regions: head and neck, trunk, pelvis and genitalia, upper extremities, and lower extremities. Classical lesions appeared predominantly on the pelvis and trunk. The solitary type appeared most frequently on the pelvis and lower extremities.

The number of published articles about NLS has increased significantly since 2012 ( Fig. 4 ). Awareness of NLS has probably increased in many countries. We conducted a literature review, for which we searched PubMed with the keyword “NLS.” The search was limited to articles written in English and whose full text was available. We analyzed the following data: year of report, nation of corresponding author, sex of patient, age at onset, duration of disease, location of lesion, type of lesion, associated symptoms, pathological findings, and treatment. Among the countries of the authors ( Fig. 5 ), India, the Republic of Korea, and the United States were most common. In total, 112 articles about 149 cases were selected from the PubMed search. Of the 149 patients, 78 were male and 71 were female. We found no sex difference in the prevalence of NLS. In 114 cases, the lesions were the classical type; in 33 cases, the solitary type. Two patients had both classical and solitary lesions ( Fig. 6 ).

The lesions were located in five regions: head and neck, trunk, pelvis and genitalia, upper extremities, and lower extremities. Classical lesions were predominantly on the pelvis and trunk, and solitary lesions were frequently on the pelvis and lower extremities ( Fig. 6 ).

**Fig. 7:**
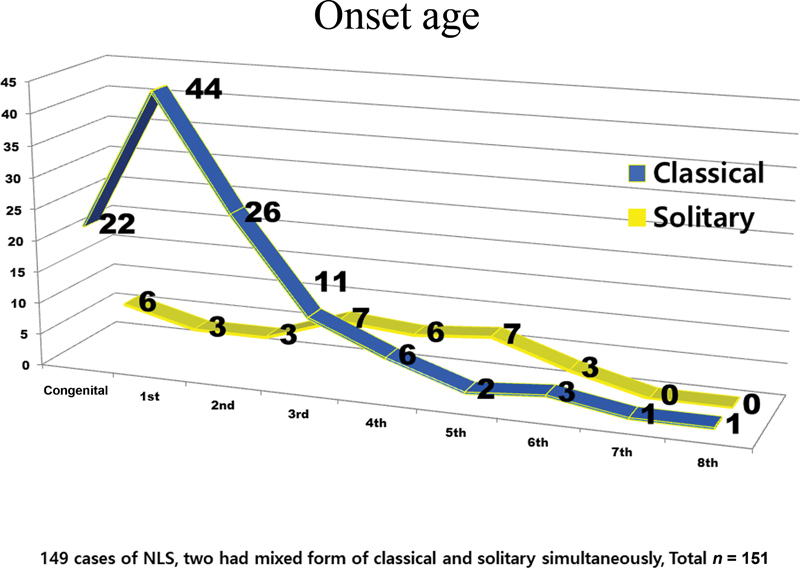
Onset of lesion. The classical type of nevus lipomatosus superficialis (NLS) usually was present at birth or appeared in the first three decades of life. The solitary type appeared in the third to fifth decades of life. In 28 cases (18%), the classical and solitary types were congenital.

With regard to onset, findings in previous reports 2 were consistent with ours: the classical type usually was present at birth or appeared in the first three decades of life, and the solitary type appeared in the third to fifth decades of life ( Fig. 7 ). In 28 patients (18%), classical and solitary lesions were congenital.

**Fig. 8:**
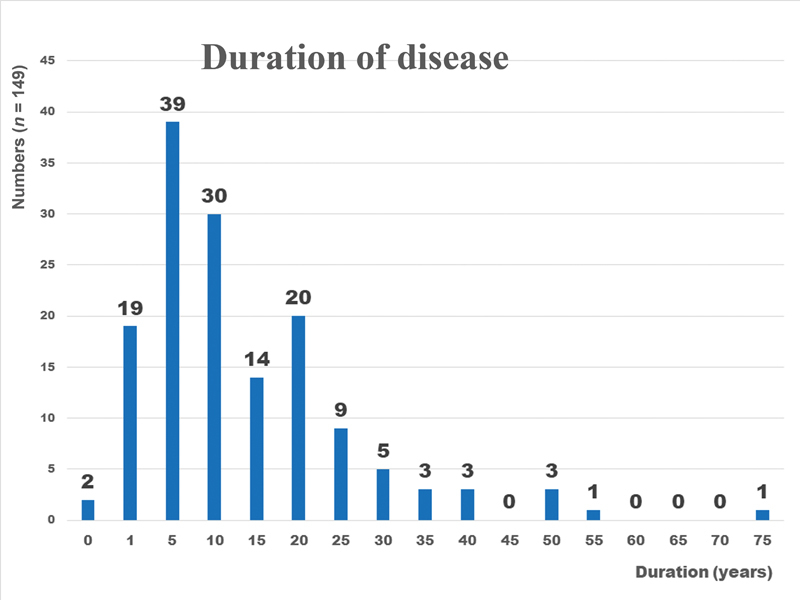
“Duration of the disease” means the time interval between the patient's awareness of the lesion's presence and the visit to the clinic. Among durations of less than 20 years, five durations were most common. This suggested that nevus lipomatosus superficialis was typically present for more than 5 years and that the lesion increased gradually in size.

“Duration of the disease” means the time interval between the patient's awareness of the lesion's presence and the visit to the clinic. Among durations of less than 20 years, five were most common ( Fig. 8 ).

## Discussion

In the United States, the Food and Drug Administration defines a rare disease as any disease that affects fewer than 200,000 Americans. In Europe, a disease is defined as rare if it affects fewer than 1 per 2,000 people. 3 Until now, no more than 200 cases of NLS were reported, according to our PubMed search; thus, NLS could have been classified as a rare disease. Data on prevalence will be required in the near future.

Three patients with NLS had relatives with the condition. 4 5 In one case of NLS, the patient had a 2p24 deletion; the authors suggested that the chromosomal abnormality played a role in NLS. 6

NLS has been described as asymptomatic. According to our literature review, however, 10 patients (6.7%) presented with symptoms such as itching, pain, foul odor, and rhinitis. One patient had dyspnea as a result of an intranasal lesion. According to another report, compressive neuropathy of the ulnar nerve was caused by firm subcutaneous nodules of thickened fibrotic dermis containing adipose tissue. 7 Systemic abnormalities and malignant changes have not been associated with NLS.

Several combinations of lesions have been reported. Comedones, abnormal hair growth or alopecia, and acanthosis most frequently accompanied NLS. Combinations of NLS with trichofolliculoma, folliculosebaceous hamartoma, or perifollicular fibroma were rare. In two cases, NLS was accompanied by intramuscular lipoma. 8 9 In one case, NLS was accompanied by a polypoid type of basal cell carcinoma of the scalp. 10 In our patient, NLS and sebaceous trichofolliculoma were present; in our literature review, we found only one report in which NLS was accompanied by a sebaceous trichofolliculoma. 11

Sebaceous trichofolliculoma has hair follicles with sebaceous gland components, which are epithelial components. However, there are two other, similar lesions that should be differentiated from sebaceous trichofolliculoma histopathologically: trichofolliculoma, which has only a follicular structure, and folliculosebaceous hamartoma, which contains not only epithelial components of follicles and sebaceous components but also mesenchymal structures of vessels, cartilage, and other tissues.

Plastic surgeons are not familiar with NLS. It is often misdiagnosed as a common skin lesion such as soft fibroma, skin tag (acrochordon), neurofibroma, or lipoma. The differential diagnosis of the classical type includes mucinous nevus, 12 sebaceous nevus, hemangioma, hairy nevus, lymphangioma, and focal dermal hyperplasia (Goltz syndrome). The solitary type is often confused with skin tags, solitary neurofibromas, and solitary lipofibromas. 13

Treatment is usually not necessary except for cosmetic reasons. Surgical resection is curative, and no recurrence has been reported. Other treatment modalities have been attempted: CO _2_ laser treatment, 14 15 cryotherapy, 16 intralesional phosphatidylcholine injection, 17 topical corticosteroid application, 18 and electrodessication. 19 However, because the lesion is large, reconstructing the defect after lesion removal is difficult. Physicians should be aware of this rare disease because early detection allows for less invasive resection of the lesion and more conservative reconstruction of the defect.
